# MultiPaths: a Python framework for analyzing multi-layer biological networks using diffusion algorithms

**DOI:** 10.1093/bioinformatics/btaa1069

**Published:** 2020-12-26

**Authors:** Josep Marín-Llaó, Sarah Mubeen, Alexandre Perera-Lluna, Martin Hofmann-Apitius, Sergio Picart-Armada, Daniel Domingo-Fernández

**Affiliations:** Department of Bioinformatics, Fraunhofer Institute for Algorithms and Scientific Computing (SCAI), Sankt Augustin 53757, Germany; B2SLab, Departament d’Enginyeria de Sistemes, Automàtica i Informàtica Industrial, Universitat Politècnica de Catalunya, CIBER-BBN, Barcelona 08028, Spain; Department of Bioinformatics, Fraunhofer Institute for Algorithms and Scientific Computing (SCAI), Sankt Augustin 53757, Germany; Fraunhofer Center for Machine Learning, Germany; B2SLab, Departament d’Enginyeria de Sistemes, Automàtica i Informàtica Industrial, Universitat Politècnica de Catalunya, CIBER-BBN, Barcelona 08028, Spain; Department of Bioinformatics, Fraunhofer Institute for Algorithms and Scientific Computing (SCAI), Sankt Augustin 53757, Germany; B2SLab, Departament d’Enginyeria de Sistemes, Automàtica i Informàtica Industrial, Universitat Politècnica de Catalunya, CIBER-BBN, Barcelona 08028, Spain; Department of Bioinformatics, Fraunhofer Institute for Algorithms and Scientific Computing (SCAI), Sankt Augustin 53757, Germany; Fraunhofer Center for Machine Learning, Germany

## Abstract

**Summary:**

High-throughput screening yields vast amounts of biological data which can be highly challenging to interpret. In response, knowledge-driven approaches emerged as possible solutions to analyze large datasets by leveraging prior knowledge of biomolecular interactions represented in the form of biological networks. Nonetheless, given their size and complexity, their manual investigation quickly becomes impractical. Thus, computational approaches, such as diffusion algorithms, are often employed to interpret and contextualize the results of high-throughput experiments. Here, we present MultiPaths, a framework consisting of two independent Python packages for network analysis. While the first package, DiffuPy, comprises numerous commonly used diffusion algorithms applicable to any generic network, the second, DiffuPath, enables the application of these algorithms on multi-layer biological networks. To facilitate its usability, the framework includes a command line interface, reproducible examples and documentation. To demonstrate the framework, we conducted several diffusion experiments on three independent multi*-omics* datasets over disparate networks generated from pathway databases, thus, highlighting the ability of multi-layer networks to integrate multiple modalities. Finally, the results of these experiments demonstrate how the generation of harmonized networks from disparate databases can improve predictive performance with respect to individual resources.

**Availability and implementation:**

DiffuPy and DiffuPath are publicly available under the Apache License 2.0 at https://github.com/multipaths.

**Supplementary information:**

Supplementary data are available at *Bioinformatics* online.

## 1 Introduction

Emergent properties of biological processes primarily arise from complex interactions linking physical entities which, in turn, can build up complex biological networks, such as metabolic, signaling and regulatory. The use of these networks has become commonplace for a variety of analytic tasks, yet integrated networks have been shown to be more robust resources for analytic usage (Huang *et al.*, 2018). Thus, several frameworks, such as Bio2RDF (Belleau *et al.*, 2008), have been proposed to facilitate the integration of these networks from heterogeneous sources.

Numerous methods for network analysis derived from graph theory have been adapted for a broad range of applications in the biomedical domain including target prioritization, gene prediction and patient stratification (Barabási *et al.*, 2011; Pai *et al.*, 2019). Amongst these methods, network propagation or diffusion, in particular, comprises a broad family of algorithms that infer node labels based on the sharing of labels through network connections (Cowen *et al.*, 2017).

Though a wide variety of algorithms exist, user-friendly software that can enable researchers to implement and compare several methods are lacking. Not only does this impede their adoption and reproducibility but it also compels researchers to re-implement the algorithms for their particular needs. While recently, the R packages diffuStats and RANKS (Picart-Armada *et al.*, 2018; Valentini *et al.*, 2016) have addressed this issue, a framework that offers a pipeline to build harmonized networks from biological databases along with an array of ready-to-use diffusion algorithms has yet to be established.

Here, we present MultiPaths, a Python framework for the analysis of multi-*omics* data by classical and statistically normalized diffusion algorithms on harmonized networks from custom or predefined selections of biological databases. We demonstrate how MultiPaths enables contextualizing multi*-omics* experiments by presenting an application scenario on multiple datasets containing transcriptomics, metabolomics and miRNomics data.

## 2 Implementation

The MultiPaths framework contains two independent Python packages: DiffuPy and DiffuPath. While DiffuPy is specifically designed for the implementation of diffusion algorithms, DiffuPath is capable of both generating harmonized biological networks, and running the algorithms over these networks. Their functionalities can be accessed programmatically and via a command line interface (CLI) for nonbioinformaticians. Their modular design eases the inclusion of network resources and algorithms in future releases.

### 2.1 DiffuPy

The first of the two packages in the framework, DiffuPy, enables propagating user-defined labels, either as lists of entities or lists of entities with their corresponding quantitative values, on a user-defined network. DiffuPy comprises four diffusion scores and five graph kernels that can be run on generic networks on different formats (Supplementary Text).

### 2.2 DiffuPath

The second package, DiffuPath, wraps the generic diffusion algorithms from DiffuPy and applies them to biological networks. To that end, DiffuPath comprises a comprehensive pipeline that extends from the generation of harmonized networks from multiple biological databases to the visualization and analysis of the diffusion results (Supplementary Text). The pipeline provides a user-friendly CLI that enables users to create customized networks from a pool of databases or predefined collections based on their input data, directly run diffusion algorithms on these networks, and analyze them in a few commands. Finally, we would like to note that, while DiffuPath already includes a wide range of databases, the framework supports the integration of any number of databases in standard network formats.

## 3 Application

To demonstrate the framework, we run various diffusion algorithms from DiffuPy on four networks corresponding to four pathway databases generated through DiffuPath. The input labels for the diffusion derive from three independent datasets containing differential entities from three *-omics* modalities: transcriptomics, metabolomics and miRNomics. The four networks consist of three well-established pathway databases: KEGG, Reactome and WikiPathways (Fabregat *et al.*, 2018; Kanehisa *et al.*, 2017; Slenter *et al.*, 2018) as well as their combined representation, PathMe (Domingo-Fernández *et al.*, 2019). Our hypothesis is that by integrating the three resources, PathMe covers a larger scale of interactions and entities as well as a broader range of interaction and modality types which can ultimately serve to improve prediction performance.

**Fig. 1.**
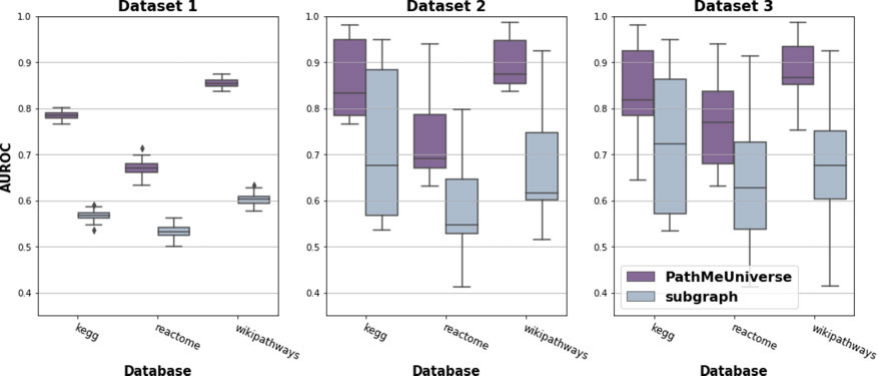
Prediction performance of raw diffusion over the integrated PathMe network and the correspondent database subgraph for three multi-*omics* datasets. Each box plot shows the distribution of the area under the ROC curves (AUROCs) over 100 repeated holdout validations. Details on the evaluation can be found in the Supplementary Text

For each of the three datasets which investigated specific biological processes, we compared the prediction performance of the various diffusion algorithms in identifying genes, metabolites and miRNAs (for details see Supplementary Text Section S4: Case scenario). This was repeated for each of the four networks and the performance was evaluated using a repeated holdout approach. For the raw diffusion scores, the distribution of area under the ROC curve (AUROC) scores indicated a significant improvement in prediction performance of the integrated multi-layer network over each of the individual databases (Fig. 1).

## 4 Discussion

This work has presented the first Python framework that implements numerous diffusion algorithms along with a pipeline to build customized harmonized networks from multiple biological databases. The importance of this integration is highlighted by our three case scenarios where a harmonized network leverages three *-omics* modalities (Di Nanni *et al.*, 2020) to increase predictivity in line with Huang *et al.* (2018). Furthermore, the integrated networks contain additional entities like biological processes and clinical readouts (e.g. symptoms and diseases), allowing a rich contextualization of the experimental readouts (Supplementary Text). This case scenario demonstrates the utility of diffusion algorithms to provide the interpretation of biological networks in the context of pathways, and thereby, elucidate the properties of biological processes underlying these networks. Additionally, users can conduct analyses from biological networks to generic networks from other fields (e.g. social media) as well as incorporate additional kernels or diffusion algorithms to DiffuPy. As a final remark, although large-scale and integrated multi-layer networks can improve prediction performance, greater computational power is required as the size of a network grows.

## Funding

This work was developed in the Fraunhofer Cluster of Excellence ‘Cognitive Internet Technologies’ and the DPI2017-89827-R, Networking Biomedical Research Centre in the subject area of Bioengineering, Biomaterials and Nanomedicine (CIBER-BBN). 

*Conflict of Interest*: none declared. 

## Supplementary Material

btaa1069_Supplementary_Data
