# Supplementary material for: MultiPaths: a Python framework for analyzing multi-layer biological networks using diffusion algorithms
Source: Bioinformatics. 2020 Dec 26;37(1):137–9. doi: 10.1093/bioinformatics/btaa1069 (PMC8034528; doi:10.1093/bioinformatics/btaa1069)
Supplement: btaa1069_Supplementary_Data [file btaa1069_supplementary_data.pdf]

Additional File

Outline

1. DiffuPy
2. DiffuPath
3. Software Design
4. Case Scenario

Supplementary Text

1. DiffuPy

DiffuPy is a Python package that has been designed to run network propagation algorithms on generic networks (**Supplementary Figure 1)**. DiffuPy implements four existing network propagation algorithms and five graph kernels, introducing them to the Python community as, until now, they were limited to other programming languages such as R (e.g., diffuStats (Picart-Armada *et al*., 2017a) and RANKS (Valentini *et al.*, 2016)). In its implementation, DiffuPy leverages NetworkX (Hagberg *et al*., 2008), one of the most commonly used packages for working with networks in Python.

Scores including **raw**, **ml** and **gm** are computed as $f = Ky$, where f is the score vector, K is the graph kernel and y contains the input scores. These scores differ on how y is codified when the input is defined as a set of positive, negative and unlabelled nodes **(Supplementary Table 1)**. A fourth score, **z**, corrects node-wise **raw** scores with an exact z-score under the permutation of labelled nodes. **z** has been found to be better suited over **raw** in the interpretation of metabolomics data using heterogeneous knowledge graphs from the KEGG database (Picart-Armada *et al.* 2017b). On the other hand, several options are available for choosing K **(Supplementary Table 2)**, which dictates how the propagation behaves. By default, the regularised Laplacian kernel is used, which is a commonly used fluid or heat propagation model (Cowen *et al*., 2017).

Furthermore, DiffuPy provides a command line interface (CLI) for improving its usability such that analyses can be conducted on the terminal, as per the guidelines in Grüning *et al*. (2019). The most recent version of the software allows users to input several standard network formats such as SIF or GraphML, facilitating its usage across different formats. This in turn also enables running the diffusion algorithms on non-biological networks from other fields, such as social media. Finally, the source code of the DiffuPy Python package is available at https://github.com/multipaths/diffupy, its latest documentation can be found at https://diffupy.readthedocs.io and its distributions can be found on PyPI at https://pypi.org/project/diffupy.

Method	y^+	y^-	y^u	Normalized	Stochastic	Quantitative	Reference
raw	1	0	0	No	No	Yes	Vandin *et al.* (2010)
ml	1	-1	0	No	No	No	Tsuda *et al.* (2010)
gm	1	-1	k	No	No	No	Mostafavi *et al.* (2008)
z	1	0	0*	Yes	No	Yes	Harchaoui *et al.* (2013)

Supplementary Table 1. Diffusion methods implemented in DiffuPy. Methods differ on how the input labels are codified in the propagation (positive entities y^+, negatives y^- and unlabelled y^u). Besides the positive/negative/unlabelled paradigm, **raw** and **z** also accept

quantitative inputs, which are passed to the propagation directly. The **z** method offers a statistical normalization, specifically an exact z-score, based on the permutation of the labelled nodes.

Kernel	Regularisation function	Reference
Regularised Laplacian	$r(\lambda) = 1 + \sigma^2\lambda$	Smola *et al.* (2003)
Diffusion process	$r(\lambda) = exp(\frac{\sigma^2}{2}\lambda)$	Smola *et al.* (2003)
p-Step random walk	$r(\lambda) = (a - \lambda)^{-p}$	Smola *et al.* (2003)
Inverse cosine	$r(\lambda) = (cos(\lambda\frac{\pi}{4}))^{-1}$	Smola *et al.* (2003)
Commute-time kernel	$r(\lambda) = \lambda$	Yen *et al.* (2007)

Supplementary Table 2. Graph kernels implemented in DiffuPy. The regularisation function is the transformation applied to the graph Laplacian spectrum, as defined in Smola (2003), that characterises each kernel. $\sigma^2 > 0$, $a \geq 2$ and $p \geq 1$ are kernel parameters.

2. DiffuPath

The primary goal of DiffuPath is to act as a connector between harmonized biological networks and the array of diffusion algorithms implemented in DiffuPy. DiffuPath leverages two resources, PathMe (Domingo-Fernández *et al*., 2019) and Bio2BEL (https://github.com/bio2bel), for the construction of biological networks. The first of these two resources, PathMe, automatically extracts various entities (e.g., genes, proteins, metabolites and biological processes) and relationships from multiple primary databases (i.e., KEGG, Reactome and WikiPathways). These entities and relationships are then transformed into a common, network-like schema (i.e., Biological Expression Language; BEL), facilitating their integration. Similarly, Bio2BEL integrates numerous resources containing structured biological knowledge and provides the necessary tools to convert these into BEL. By leveraging these two resources, DiffuPath offers a pipeline that can generate harmonized networks based on the database selected by the user **(Supplementary Figure 1)**. In total, users can select from nine single-resource databases **(Supplementary Table 3)**. By using the harmonized network of all available databases, one could cover up to three *-omics* modalities (i.e., genomics/proteomics, miRNAs, metabolomics) as well as clinical endpoints (i.e., symptoms, side effects), biological processes (i.e., pathways) and diseases. To facilitate users choosing a selection, we also provide predefined collections of databases based on the goal of the study and dataset characteristics **(Supplementary Table 4)**. Furthermore, DiffuPath contains functions to explore the overlap between the input and the network in the *views* module. Finally, the source code is available at https://github.com/multipaths/diffupath, its latest documentation can be found at https://diffupath.readthedocs.io, and its distributions can be found on PyPI at https://pypi.org/project/diffupath.

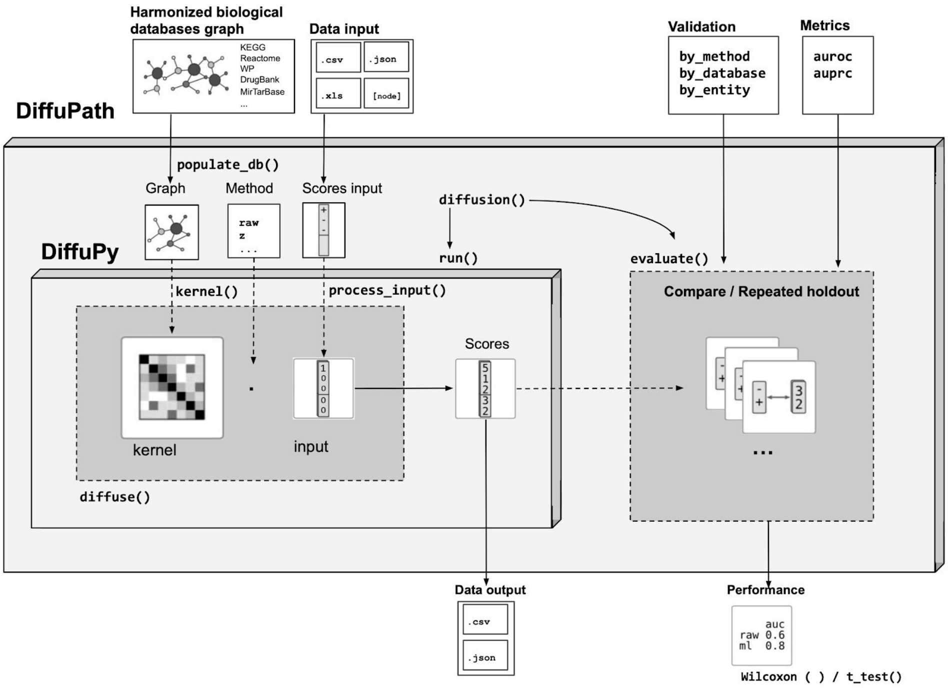

Supplementary Figure 1. DiffuPath and DiffuPy framework.

Name	Description	Reference
DDR	Disease-disease associations	Menche *et al.* (2015)
DrugBank	Interactions between drugs and drug targets with over 10,000 drugs	Wishart *et al.* (2018)
Gene Ontology (GO)	Flexible hierarchy of tens of thousands of biological processes	Ashburner *et al.* (2000)
HSDN	Associations between thousands of diseases with hundreds of symptoms	Zhou *et al.* (2014)
KEGG	Multi-omics interactions present in hundreds of biological pathways	Kanehisa *et al.* (2016)
MirTarBase	Nearly 300,000 experimentally validated interactions between miRNA and their targets	Huang *et al.* (2020)
Reactome	Multi-*omics* interactions present in thousands of biological pathways	Fabregat *et al.* (2018)
SIDER	Associations between over a thousand drugs and side effects	Kuhn *et al.* (2016)
WikiPathways	Multi-*omics* interactions present in hundreds of biological pathways	Slenter *et al.* (2017)

Supplementary Table 3. **List of biological databases available from DiffuPath.**

Collection	Databases	Description
#1	KEGG, Reactome, and WikiPathways	*-omics* and biological processes/pathways
#2	KEGG, Reactome, WikiPathways, and DrugBank	*-omics* and biological processes/pathways with a strong focus on drug/chemical interactions
#3	KEGG, Reactome, WikiPathways, and MirTarBase	*-omics* and biological processes/pathways enriched with miRNAs

Supplementary Table 4. **Predefined collections available in DiffuPath.**

Database	Total Nodes	Gene	Protein	RNA	miRNA	Abundance	Biological Process	Pathology	Complex	Composite
DDR	296	-	-	-	-	-	-	296	-	-

DrugBank	8355	-	2802	-	-	5553	-	-	-	-
GO	33577	-	-	-	-	2078	29393	-	2106	-
HSDN	433	-	-	-	-	-	-	433	-	-
KEGG	13465	-	8254	409	-	4452	461	-	-	38
MirTarBase	26527	-	-	22636	3891	-	-	-	-	-
Reactome	22407	563	8585	147	2	2751	2218	-	8135	6
SIDER	8497	-	-	-	-	1507	-	6990	-	-
WikiPathways	4243	2933	472	42	-	796	-	-	-	-

Supplementary Table 5. Statistics of the entity types for each of the individual databases available in DiffuPath.

Collection	Total Nodes	Gene	Protein	RNA	miRNA	Abundance	Biological Process	Pathology	Complex	Composite	Reaction
#1	60476	15229	10707	151	-	7314	2679	-	9184	1620	13592
#2	68831	15229	13509	151	-	12867	2679	-	9184	1620	13592
#3	87003	15229	10707	22787	3891	7314	2679	-	9184	1620	13592

Supplementary Table 6. Statistics of the entity types for each of the collections available in DiffuPath.

3. Software Design

Both DiffuPy and DiffuPath have a tool chain consisting of pytest (https://github.com/pytest-dev/pytest) as a testing framework, coverage (https://github.com/nedbat/coveragepy) to assess testing coverage, sphinx (https://github.com/sphinx-doc/sphinx) to build documentation, flake8 (https://github.com/PyCQA/flake8) to enforce code and documentation quality, setuptools (https://github.com/pypa/setuptools) to build distributions, pyroma (https://github.com/regebro/pyroma) to enforce package metadata standards and tox (https://github.com/tox-dev/tox) as a build tool to facilitate the usage of each of these tools in a reproducible way. These packages leverage community and open source resources to improve their usability by using Travis-CI (https://travis-ci.com) as a continuous integration service, monitoring testing coverage with Codecov (https://codecov.io) and hosting its documentation on Read the Docs (https://readthedocs.org).

4. Case Scenario

4.1. Network

The harmonized network chosen for the case scenario comprises three major pathway databases (i.e., KEGG; Kanehisa *et al.*, 2016, Reactome; Fabregat *et al.*, 2018, and WikiPathways; Slenter *et al.*, 2017). This integrated network was selected as it comprises pathway information from three highly-cited and open-sourced databases, covers a broad spectrum of human pathways, and incorporates three -*omics* modalities, thus enabling enhanced predictive power. The harmonized network of these three resources was generated using the CLI of DiffuPath leveraging its close integration with PathMe (Domingo-Fernández *et al.*, 2019). Because DiffuPath permits the separation of modalities, diffusion scores can be stratified by node type. For the case scenario, we thus selected PathMe as it incorporates multiple entity types (e.g., genes, miRNAs, and metabolites) that are also found in the multi-*omics* datasets chosen. Although edges can have attributes and directionality, the four networks used for label propagation were regarded as simple graphs (i.e., undirected, unweighted, no multi-edges or loops).

The statistics, including the number and type of nodes and edges of individual pathway database networks that DiffuPath provides are displayed in **Supplementary Table 5.** Group nodes, such as protein complexes and protein families, were represented through a group node formalism in which groups of entities were denoted as

individual nodes and were connected to their neighbours by single edges. By selecting this representation approach, we sought to preserve biological context. Furthermore, we performed two additional preprocessing steps for the PathMe network. Firstly, isolated nodes were removed from the network. Secondly, if any proteins, RNAs and/or genes were equivalent, these nodes were collapsed into a single node (i.e., gene). The difference in statistics between collapsed and non-collapsed nodes are presented in **Supplementary Table 6 and Supplementary Figure 2**, respectively.

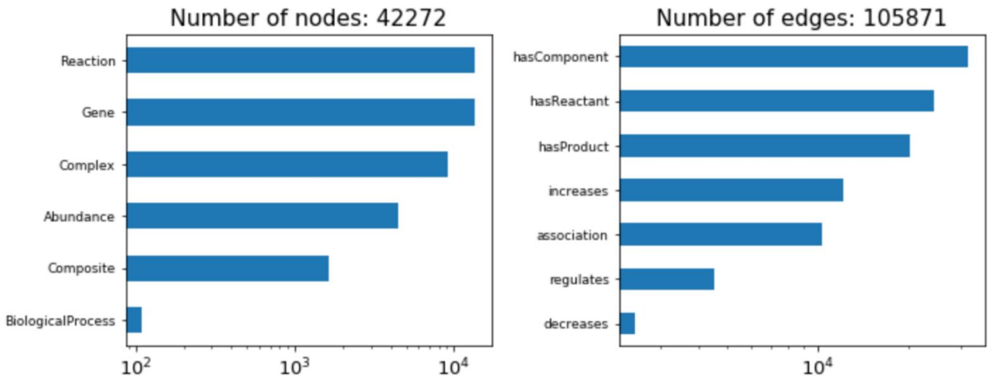

Supplementary Figure 2. Node and edge statistics of the PathMe network.

4.2. Datasets

In selecting the multi-*omics* datasets used in the case scenario for DiffuPath validation, the following criteria were applied:

- Each dataset had to contain three *-omics* modalities: molecular readouts on genes, metabolites and miRNAs.
- Each dataset had to be derived from human experiments.
- The original publication had to include differential analysis in order to avoid any bias in the preprocessing steps as well as include biological interpretation for validation purposes.

Following these criteria, three datasets were obtained and each was downloaded and customly parsed using DiffuPy processing tools. Data from the datasets was then mapped to the network, details of which are given below.

The **first dataset** comprises multi *-omics* experiments aimed to investigate the effect of exposure to cyclosporin A in hepatic cells (Van den Hof *et al.,* 2015). The authors quantified gene and miRNA expression as well as metabolomics and integrated the three *-omics* modalities to conduct an integrated pathway analysis. **Supplementary Figure 3** shows the overlap of each of these three modalities while **Supplementary Figure 4** depicts distributions of input measurements by entity type.

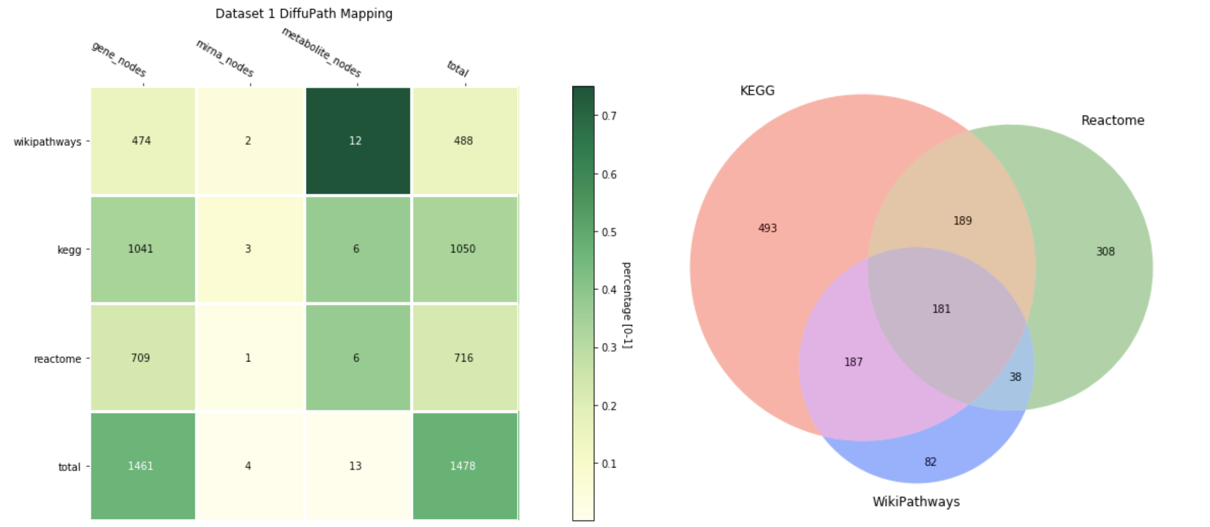

Supplementary Figure 3. Mapping between dataset 1 and the network, stratified by entity modality (x axis) and by database (y axis) (left). The Venn diagram explores the overlap between multiple databases for the mapped entities (right). Colours in each cell of the heatmap represent the percent coverage of each modality for each database, except for the last row, where the denominator is the total amount of original entities in the dataset. The number in each cell corresponds to the total number of entities of a specific modality after mapping the input. The final column of the heatmap denotes the total number of entities in each of the three databases and the bottom row corresponds to the sum total of each modality across databases. Stratification by modality was performed in order to prevent bias of the results towards the most frequently occurring entity.

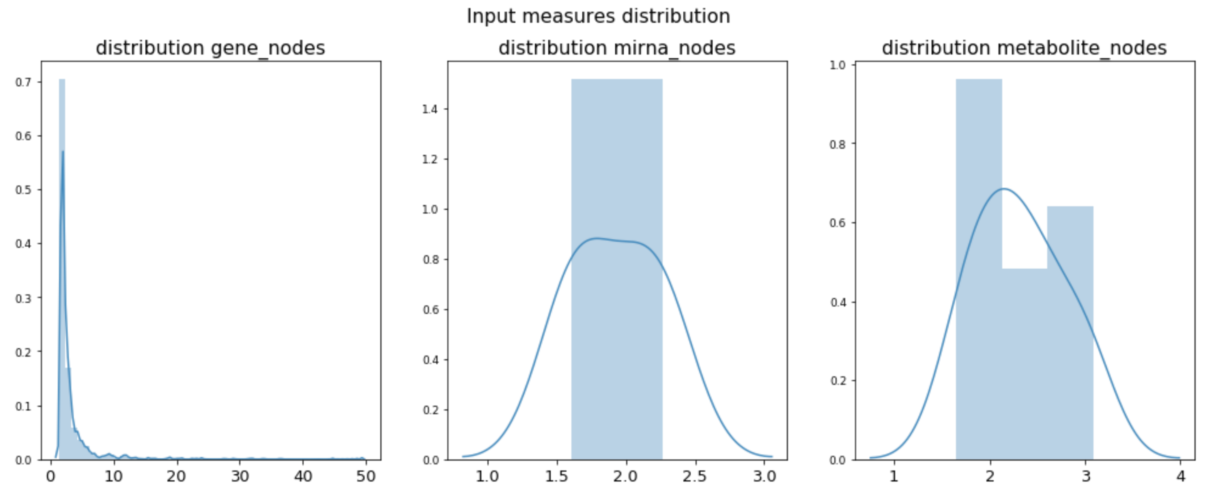

Supplementary Figure 4. Distribution of the selected input measurements (i.e., absolute $\log_2$ fold changes) for dataset 1 stratified by -*omics* modality. Negative and positive raw values have been normalized by calculating their absolute value since the results of the diffusion need to be sorted in order to calculate the AUC values. The distribution of genes is relatively dispersed compared to the uniform distribution of miRNAs and metabolites. This can be attributed to the successful mapping of a larger proportion of genes than miRNAs or metabolites, thus resulting in a larger variance.

The **second dataset** aimed at identifying major metabolic pathways and potential biomarkers involved in prostate cancer (Ren *et al.,* 2016). Relatively fewer measurements of gene expression and metabolites were taken for this dataset, from which pathway information was inferred. **Supplementary Figure 5** shows the overlap of each of these two modalities

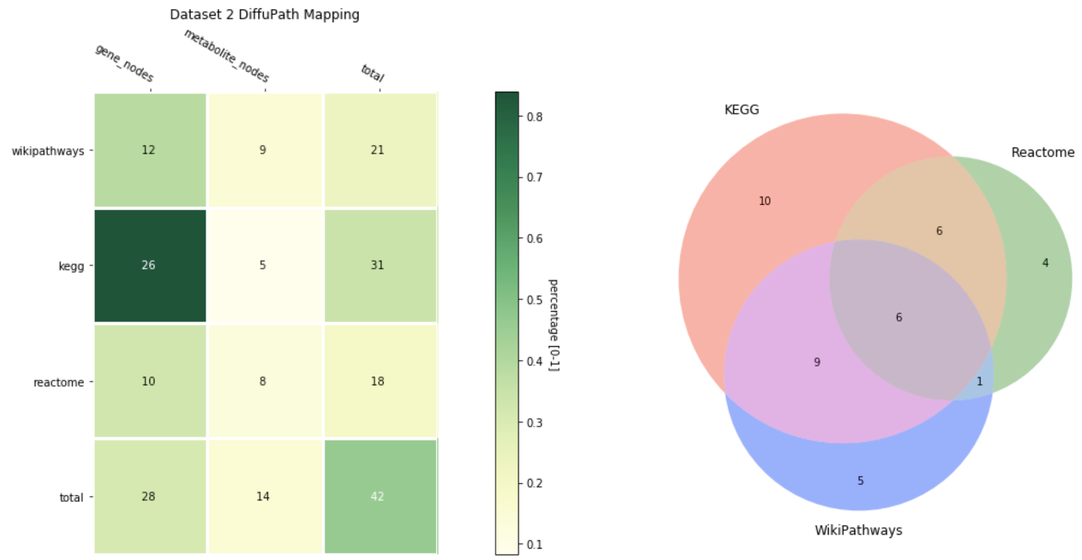

Supplementary Figure 5. Mapping between dataset 2 and the network, stratified by entity modality (x axis) and by database (y axis) (left). The Venn diagram explores the overlap between the databases for the mapped entities (right). Colours in each cell of the heatmap represent the percent coverage of each modality for each database, except for the last row, where the denominator is the total amount of original entities in the dataset. The number in each cell corresponds to the total number of entities of a specific modality after mapping the input. The final column of the heatmap denotes the total number of entities in each of the three databases and the bottom row corresponds to the sum total of each modality across databases, Stratification by modality was performed in order to prevent bias of the results towards the most frequently occurring entity.

The **third dataset** investigates the carcinogenic mechanisms that differentiate intrahepatic cholangiocarcinoma from hepatocellular carcinoma (Murakami *et al.,* 2015). This dataset contains several genes, metabolites and one miRNA, as shown in **Supplementary Figure 7.**

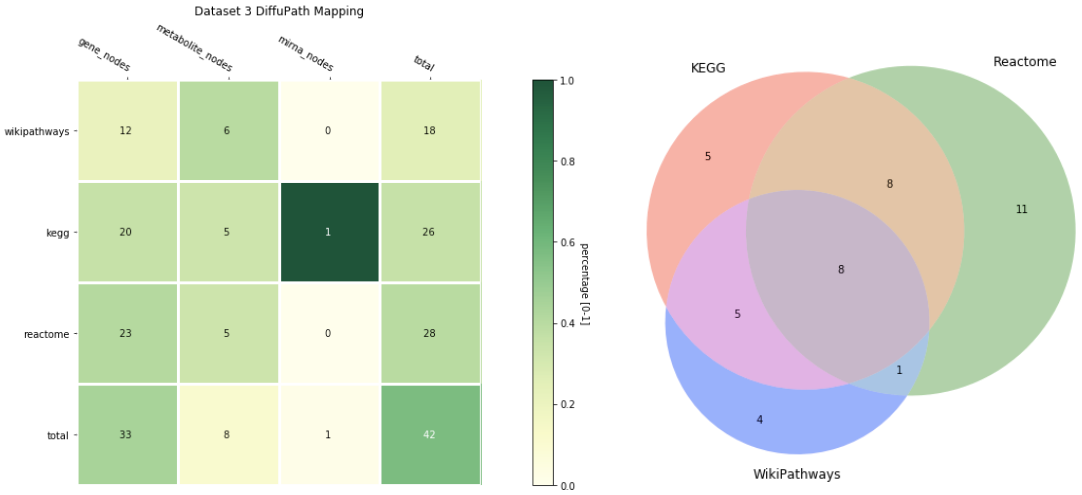

Supplementary Figures 6. Mapping between dataset 3 and the network, stratified by entity modality (x axis) and by database (y axis) (left). The Venn diagram explores the overlap between the databases for the mapped entities (right). Colours in each cell of the heatmap represent the percent coverage of each modality for each database, except for the last row, where the denominator is the total amount of original entities in the dataset. The number in each cell corresponds to the total number of entities of a specific modality after mapping the input. The final column of the heatmap denotes the total number of entities in each of the three databases and the bottom row corresponds to the sum total of each modality across databases, Stratification by modality was performed in order to prevent bias of the results towards the most frequently occurring entity.

4.3. Diffusion evaluation specifications

Network propagation has been previously applied to knowledge graphs and has been shown to benefit from statistical normalization in pathway analysis on heterogeneous networks. Raw and normalized z diffusion scores implemented in diffuStats (Picart-Armada *et al*., 2017a) were adapted into DiffuPy, giving the user the flexibility to select the statistical background of the analysis (choosing between z normalization or other diffusion procedures lacking statistical normalization). Raw and z normalization label diffusion were compared (see "evaluation by method") together with two baselines: i) a random prioritiser as an absolute baseline, and ii) PageRank as an input-naïve prioritiser to assess how predictive centrality is. The comparison of the performance between these four methods was used to assess the predictive power of the label diffusion over three datasets previously introduced and a given network corresponding to an individual pathway database (i.e., KEGG, Reactome, and WikiPathways) or the harmonized integrative network (i.e., PathMe).

As an additional study (see "evaluation by database"), we evaluated whether PathMe, the integrative resource, yields better results than individual pathway databases. To conduct a fair comparison, diffusion algorithms were run on each of the four networks exclusively using entities from the datasets that successfully overlapped with each network. We would like to note that the PathMe network was run using the same input as the individual database it was compared against (i.e., only entities mapped to the individual database were used as an input for PathMe, although the PathMe network could have covered a larger number of entities).

In both evaluations (by method and by database), the input vectors for the propagation were provided as quantitative values for fold-change (in dataset 1) and binarized *p*-value by significance (in dataset 2 and 3). For dataset 1, vectors were codified as the absolute value of the fold change for differentially expressed entities (p-value<0.05) while for datasets 2 and 3, these entities were exclusively codified with +1 labels. Subsequently, for each of the three datasets, non-differential entities were given a 0 label and entities not measured remained unlabelled. Normalized scores rely on a permutation analysis that only shuffles labelled nodes. For each entity type, the fold-change (dataset 1) as well as the binarized labels (dataset 2 and 3) were repeatedly split into diffuse-train and validation (50% in each group). Positive labels in diffuse-train were used as an input for the diffusion procedure, whereas those in validation were used to compute the performance metrics. To ensure the most commonly occurring differential entities (e.g. genes) would not dominate over the others, a dedicated diffusion process was carried out stratifying each entity type (section 4.4.3).

After propagation, nodes were prioritised by decreasing scores after calculating their absolute values. The performance metrics used to evaluate the diffusion results were area under the ROC curve (AUROC), as a classical indicator of the overall performance for binary classifiers, and area under the precision-recall curve (AUPRC), as a measure of early retrieval. Finally, Wilcoxon tests were conducted in order to assess the differences in performance between database/method.

We would like to note the computational power required to compute and store the kernel matrix as the main limiting step while running diffusion experiments. This was exacerbated by the large number of experiments conducted to evaluate the robustness of our results (i.e., 100 repeated holdout validations for each input + network + diffusion algorithm combination). As an illustration, the kernel matrix for the PathMe network was nearly 30 GB in size and super computing nodes were required for its calculation (see Subsection 4.5 on hardware). Thus, we have made pre-calculated kernels available for download at https://github.com/multipaths/DiffuPath. Furthermore, our methodology does not take edge directionality or weights into account, which could be addressed in the future through the implementation of alternative diffusion algorithms. Finally, given the organization of the datasets and their heterogeneity, a preprocessing pipeline was implemented to extract the input data (i.e., entity names, fold changes, and/or *p*-values). This step can require a substantial amount of work since one must map and harmonize entity names from the dataset to the network as well as extract and reorganize the relevant information to a proper data structure.

4.4. Results

In this section, we discuss the results of the three validations previously introduced: i) by method, ii) by database, and iii) by entity. All figures presented in this paper and complementary analyses can be reproduced with the Jupyter notebooks located at github.com/multipaths/Results/notebooks/evaluations/repeated_holdout**.**

The evaluation by diffusion method **(Supplementary Figures 7 to 10)** shows that z normalization yields slightly better performance over raw propagation in dataset 1 using AUROC as a metric, and in dataset 2 and 3 using AUPRC as a metric. However, the Wilcoxon test does not confirm a significant difference when comparing the results of z and raw for dataset 1 for AUPRC, and dataset 2 and 3 for AUROC. When comparing diffusion methods over baselines (i.e., random and PageRank), we can see a significant difference favouring diffusion methods through the range of experiments, thus, validating the predictive power of those approaches on the integrated PathMe network. When comparing the two baselines, PageRank shows slightly better predictive power than random in dataset 2 and 3, suggesting that the differential entities from those datasets tend to be central nodes, while far greater predictive power is achieved by diffusion methods.

The second validation (by database) shows that integrating multiple databases improves performance over individual resources **(Supplementary Figures 11 to 14)**. The results of the validation show that AUROC metrics are higher in the PathMe network for all three datasets. Taking into account the differential mapping of entities for the three individual datasets **(Supplementary Figures 3, 5 and 6)**, DiffuPath improved the coverage over any single-resource network by 40-50% and therefore enhanced the ability of network propagation algorithms to correctly identify genes, metabolites and miRNAs by 10-20%, in terms of AUROC. However, this pattern does not appear when the AUPRC is used as a metric; in these cases, KEGG and Wikipathways outperform PathMe across all three datasets (though Reactome deviates from this trend which may be due to the relatively larger size of the Reactome network in comparison to the KEGG and WikiPathways ones). In such a scenario, the early retrieval performance in smaller networks (e.g. database sub-networks) is more accurate than in larger ones (e.g. integrated PathMe network). This can be attributed to a larger coverage of entities in PathMe which brings down the overall proportion of mapped entities, also leading to lower AUPRCs. For instance, the KEGG dataset includes 6,048 unique HGNC symbols while PathMe contains 13,282 unique HGNC symbols. The number of mapped genes in the first dataset are 1,041 for KEGG and 1,461 for PathMe, which is equivalent to a proportion of 17.2% in KEGG and 11.0% for PathMe, respectively. The lower proportion of mapped entities in PathMe will therefore lower the expected AUPRC.

Finally, we stratified each of the previous experiments by entity to assess the influence of each entity type (i..e, genes, metabolites, and miRNAs) on performance **(Supplementary Figures 15 and 16)**. We found that genes, which represent the largest entity type, were most influential on propagation in all three datasets in terms of AUROC/AUPRC, in contrast to either metabolites or miRNAs. However, because each of these modalities is disproportionately represented and mapped to the network, the predictive power of any one entity type over another remains inconclusive. Nonetheless, it appears that with a greater number of successfully mapped inputs of any entity type, predictive performance is likewise enhanced.

4.4.1. Validation by Method

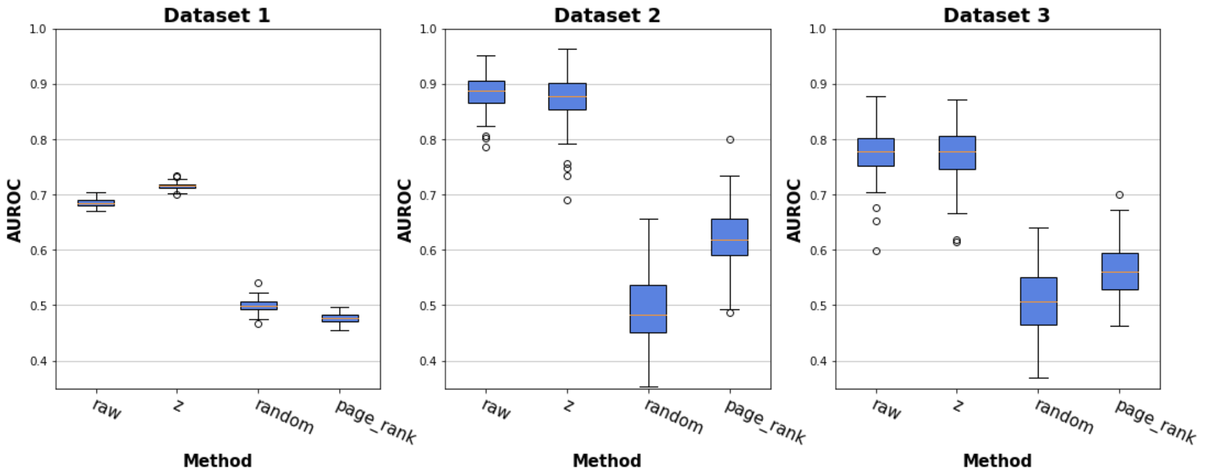

Supplementary Figure 7. Comparison of prediction performance of diffusion algorithms over the PathMe network using three different multi-omics datasets, and applying different diffusion methods, in order to validate the z-normalization performance over raw diffusion and two baselines, Random and PageRank, this second as a measure of centrality. Each box plot shows the distribution of the AUROC over 100 repeated holdout validations. This figure can be reproduced with the Jupyter notebook located in the git repository at /evaluations/repeated_holdout/by_method.

Dataset	Comparison AUROC	p_value	Significant difference
Dataset 1	('raw', 'z')	3.89656e-18	True
Dataset 1	('raw', 'random')	3.89656e-18	True
Dataset 1	('raw', 'page_rank')	3.89656e-18	True
Dataset 1	('z', 'random')	3.89656e-18	True
Dataset 1	('z', 'page_rank')	3.89656e-18	True
Dataset 1	('random', 'page_rank')	8.77788e-18	True
Dataset 2	('raw', 'z')	0.0040036	True
Dataset 2	('raw', 'random')	3.89656e-18	True
Dataset 2	('raw', 'page_rank')	3.89656e-18	True
Dataset 2	('z', 'random')	3.89656e-18	True
Dataset 2	('z', 'page_rank')	3.89656e-18	True
Dataset 2	('random', 'page_rank')	1.79393e-17	True
Dataset 3	('raw', 'z')	0.715513	False
Dataset 3	('raw', 'random')	3.89656e-18	True
Dataset 3	('raw', 'page_rank')	3.89656e-18	True
Dataset 3	('z', 'random')	3.89656e-18	True
Dataset 3	('z', 'page_rank')	4.67068e-18	True
Dataset 3	('random', 'page_rank')	7.47788e-09	True

Supplementary Figure 8. Wilcoxon test to formalize the differential comparisons of prediction performance of diffusion algorithms for the by method validation of AUROC metrics. These results can be reproduced with the Jupyter notebook located in the git repository at /evaluations/repeated_holdout/by_method.

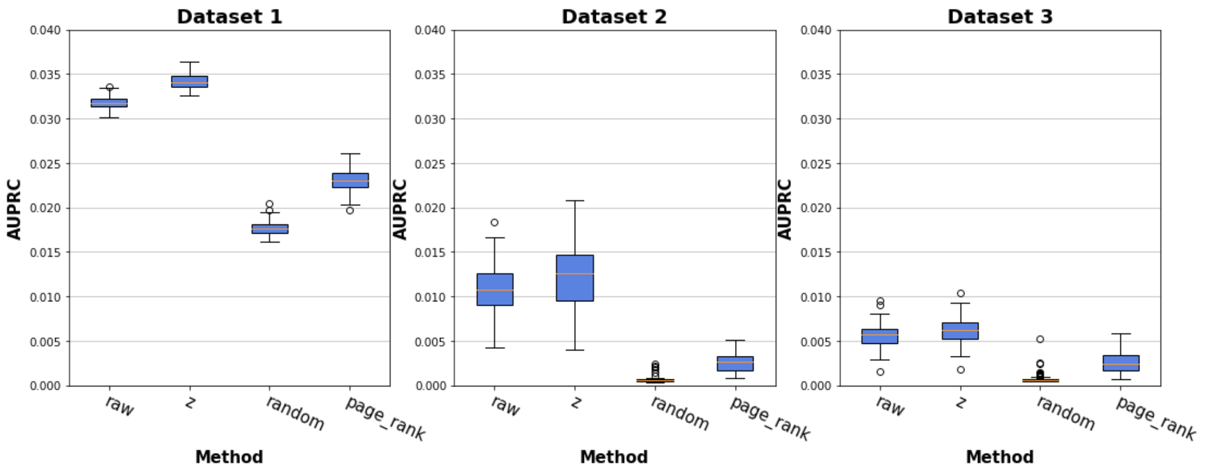

Supplementary Figure 9. Comparison of prediction performance of diffusion algorithms over PathMe network using three different multi-omics datasets, and applying different diffusion methods, in order to validate the z-normalization performance over the raw diffusion and the two baselines Random and PageRank, the latter of which was used as a centrality measure. Each box plot shows the distribution of the AUPRC over 100 repeated holdout validations. This figure can be reproduced with the Jupyter notebook located in the git repository at /evaluations/repeated_holdout/by_method.

Dataset	Comparison AUPRC	p_value	Significant difference
Dataset 1	('raw', 'z')	3.89656e-18	True
Dataset 1	('raw', 'random')	3.89656e-18	True
Dataset 1	('raw', 'page_rank')	3.89656e-18	True
Dataset 1	('z', 'random')	3.89656e-18	True
Dataset 1	('z', 'page_rank')	3.89656e-18	True
Dataset 1	('random', 'page_rank')	3.89656e-18	True
Dataset 2	('raw', 'z')	3.69817e-14	True
Dataset 2	('raw', 'random')	3.89656e-18	True
Dataset 2	('raw', 'page_rank')	3.89656e-18	True
Dataset 2	('z', 'random')	3.89656e-18	True
Dataset 2	('z', 'page_rank')	3.89656e-18	True
Dataset 2	('random', 'page_rank')	6.50411e-18	True
Dataset 3	('raw', 'z')	9.09704e-11	True
Dataset 3	('raw', 'random')	3.89656e-18	True
Dataset 3	('raw', 'page_rank')	1.05019e-17	True
Dataset 3	('z', 'random')	3.89656e-18	True
Dataset 3	('z', 'page_rank')	4.53186e-18	True
Dataset 3	('random', 'page_rank')	3.37405e-16	True

Supplementary Figure 10. Wilcoxon test to formalize the differential comparisons of prediction performance of diffusion algorithms for the by method validation of AUPRC metrics. These results can be reproduced with the Jupyter notebook located in the git repository at /evaluations/repeated_holdout/by_method.

4.4.2. Validation by Database

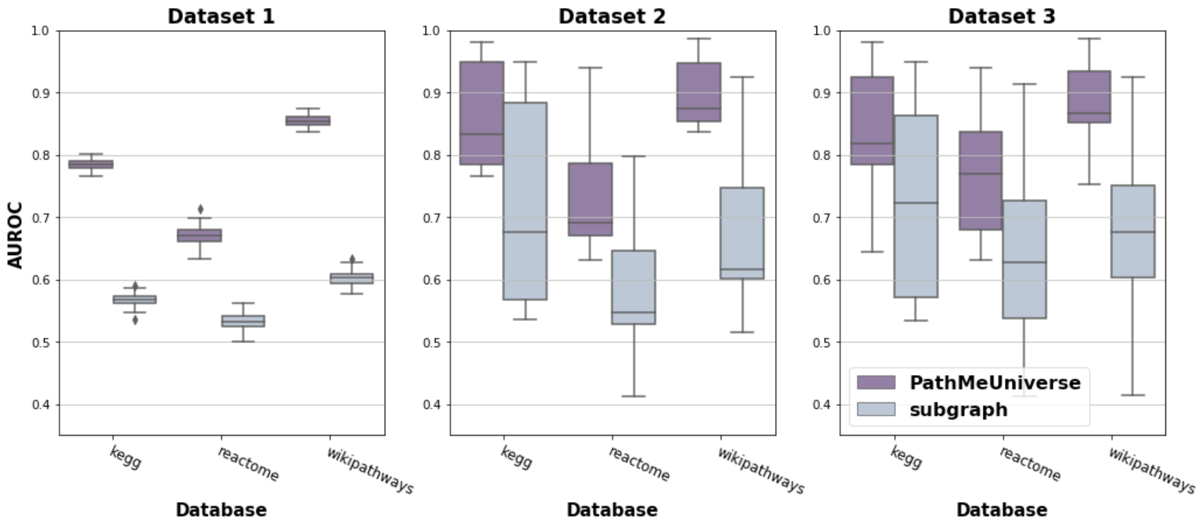

Supplementary Figure 11. Prediction performance of raw diffusion using three different multi-omics datasets over the integrated PathMe network (purple) and individual pathway databases (blue) in order to validate the PathMe network performance over single-resource pathway databases. Each box plot shows the distribution of the AUROC over 100 repeated holdout validations. This figure can be reproduced with the Jupyter notebook located in the git repository at /evaluations/repeated_holdout/by_database.

Dataset	Comparison AUROC	p_value	Significant difference
Dataset 1	('kegg_on_PathMeUniverse', 'reactome_on_PathMeUniverse')	3.89656e-18	True
Dataset 1	('kegg_on_PathMeUniverse', 'wikipathways_on_PathMeUniverse')	3.89656e-18	True
Dataset 1	('kegg_on_PathMeUniverse', 'kegg_on_subgraph')	3.89656e-18	True
Dataset 1	('kegg_on_PathMeUniverse', 'reactome_on_subgraph')	3.89656e-18	True
Dataset 1	('kegg_on_PathMeUniverse', 'wikipathways_on_subgraph')	3.89656e-18	True
Dataset 1	('reactome_on_PathMeUniverse', 'wikipathways_on_PathMeUniverse')	3.89656e-18	True
Dataset 1	('reactome_on_PathMeUniverse', 'kegg_on_subgraph')	3.89656e-18	True
Dataset 1	('reactome_on_PathMeUniverse', 'reactome_on_subgraph')	3.89656e-18	True
Dataset 1	('reactome_on_PathMeUniverse', 'wikipathways_on_subgraph')	3.89656e-18	True
Dataset 1	('wikipathways_on_PathMeUniverse', 'kegg_on_subgraph')	3.89656e-18	True
Dataset 1	('wikipathways_on_PathMeUniverse', 'reactome_on_subgraph')	3.89656e-18	True
Dataset 1	('wikipathways_on_PathMeUniverse', 'wikipathways_on_subgraph')	3.89656e-18	True
Dataset 1	('kegg_on_subgraph', 'reactome_on_subgraph')	4.67068e-18	True
Dataset 1	('kegg_on_subgraph', 'wikipathways_on_subgraph')	3.89656e-18	True
Dataset 1	('reactome_on_subgraph', 'wikipathways_on_subgraph')	3.89656e-18	True
Dataset 2	('kegg_on_PathMeUniverse', 'reactome_on_PathMeUniverse')	4.13937e-18	True
Dataset 2	('kegg_on_PathMeUniverse', 'wikipathways_on_PathMeUniverse')	0.956128	False
Dataset 2	('kegg_on_PathMeUniverse', 'kegg_on_subgraph')	2.92604e-16	True
Dataset 2	('kegg_on_PathMeUniverse', 'reactome_on_subgraph')	3.89656e-18	True
Dataset 2	('kegg_on_PathMeUniverse', 'wikipathways_on_subgraph')	3.89656e-18	True
Dataset 2	('reactome_on_PathMeUniverse', 'wikipathways_on_PathMeUniverse')	3.89656e-18	True
Dataset 2	('reactome_on_PathMeUniverse', 'kegg_on_subgraph')	9.03543e-14	True
Dataset 2	('reactome_on_PathMeUniverse', 'reactome_on_subgraph')	3.88952e-16	True
Dataset 2	('reactome_on_PathMeUniverse', 'wikipathways_on_subgraph')	0.000231007	True
Dataset 2	('wikipathways_on_PathMeUniverse', 'kegg_on_subgraph')	4.09513e-17	True
Dataset 2	('wikipathways_on_PathMeUniverse', 'reactome_on_subgraph')	3.89656e-18	True
Dataset 2	('wikipathways_on_PathMeUniverse', 'wikipathways_on_subgraph')	3.89656e-18	True
Dataset 2	('kegg_on_subgraph', 'reactome_on_subgraph')	3.89656e-18	True
Dataset 2	('kegg_on_subgraph', 'wikipathways_on_subgraph')	1.69063e-17	True
Dataset 2	('reactome_on_subgraph', 'wikipathways_on_subgraph')	1.02099e-12	True
Dataset 3	('kegg_on_PathMeUniverse', 'reactome_on_PathMeUniverse')	0.0533177	False
Dataset 3	('kegg_on_PathMeUniverse', 'wikipathways_on_PathMeUniverse')	1.85304e-09	True
Dataset 3	('kegg_on_PathMeUniverse', 'kegg_on_subgraph')	5.87765e-13	True
Dataset 3	('kegg_on_PathMeUniverse', 'reactome_on_subgraph')	3.76959e-08	True
Dataset 3	('kegg_on_PathMeUniverse', 'wikipathways_on_subgraph')	1.18577e-12	True
Dataset 3	('reactome_on_PathMeUniverse', 'wikipathways_on_PathMeUniverse')	5.3052e-06	True
Dataset 3	('reactome_on_PathMeUniverse', 'kegg_on_subgraph')	6.47331e-16	True
Dataset 3	('reactome_on_PathMeUniverse', 'reactome_on_subgraph')	2.88454e-12	True
Dataset 3	('reactome_on_PathMeUniverse', 'wikipathways_on_subgraph')	2.61031e-16	True
Dataset 3	('wikipathways_on_PathMeUniverse', 'kegg_on_subgraph')	1.082e-17	True
Dataset 3	('wikipathways_on_PathMeUniverse', 'reactome_on_subgraph')	2.87824e-17	True
Dataset 3	('wikipathways_on_PathMeUniverse', 'wikipathways_on_subgraph')	1.84789e-17	True
Dataset 3	('kegg_on_subgraph', 'reactome_on_subgraph')	0.00475967	True
Dataset 3	('kegg_on_subgraph', 'wikipathways_on_subgraph')	0.139279	False
Dataset 3	('reactome_on_subgraph', 'wikipathways_on_subgraph')	7.68318e-05	True

Supplementary Figure 12. Wilcoxon test to formalize the differential comparisons of prediction performance of diffusion algorithms for the database validation using AUROC as a metric. These results can be reproduced with the Jupyter notebook located in the git repository at /evaluations/repeated_holdout/by_database.

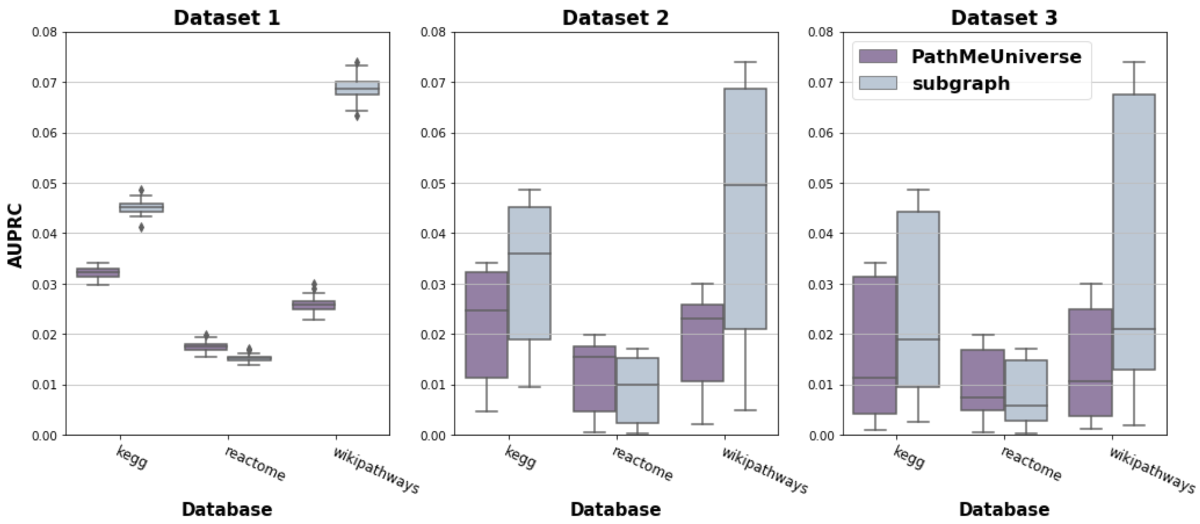

Supplementary Figure 13. Prediction performance of diffusion using three different multi-omics datasets over the integrated PathMe network (purple) and individual pathway databases (blue) in order to validate the PathMe network performance over single-resource pathway databases. Each box plot shows the distribution of the AUPRC over 100 repeated holdout validations. This figure can be reproduced with the Jupyter notebook located in the git repository at /evaluations/repeated_holdout/by_database.

Dataset	Comparison AUPRC	p_value	Significant difference
Dataset 1	('kegg_on_PathMe', 'reactome_on_PathMe')	3.89656e-18	True
Dataset 1	('kegg_on_PathMe', 'wikipathways_on_PathMe')	3.89656e-18	True
Dataset 1	('kegg_on_PathMe', 'kegg_on_subgraph')	3.89656e-18	True
Dataset 1	('kegg_on_PathMe', 'reactome_on_subgraph')	3.89656e-18	True
Dataset 1	('kegg_on_PathMe', 'wikipathways_on_subgraph')	3.89656e-18	True
Dataset 1	('reactome_on_PathMe', 'wikipathways_on_PathMe')	0.120155	False
Dataset 1	('reactome_on_PathMe', 'kegg_on_subgraph')	3.89656e-18	True
Dataset 1	('reactome_on_PathMe', 'reactome_on_subgraph')	8.13971e-14	True
Dataset 1	('reactome_on_PathMe', 'wikipathways_on_subgraph')	3.89656e-18	True
Dataset 1	('wikipathways_on_PathMe', 'kegg_on_subgraph')	3.89656e-18	True
Dataset 1	('wikipathways_on_PathMe', 'reactome_on_subgraph')	2.48991e-12	True
Dataset 1	('wikipathways_on_PathMe', 'wikipathways_on_subgraph')	3.89656e-18	True
Dataset 1	('kegg_on_subgraph', 'reactome_on_subgraph')	3.89656e-18	True
Dataset 1	('kegg_on_subgraph', 'wikipathways_on_subgraph')	3.89656e-18	True
Dataset 1	('reactome_on_subgraph', 'wikipathways_on_subgraph')	3.89656e-18	True
Dataset 2	('kegg_on_PathMe', 'reactome_on_PathMe')	5.02646e-17	True
Dataset 2	('kegg_on_PathMe', 'wikipathways_on_PathMe')	0.0461271	False
Dataset 2	('kegg_on_PathMe', 'kegg_on_subgraph')	2.29208e-14	True
Dataset 2	('kegg_on_PathMe', 'reactome_on_subgraph')	3.89656e-18	True
Dataset 2	('kegg_on_PathMe', 'wikipathways_on_subgraph')	8.13971e-14	True
Dataset 2	('reactome_on_PathMe', 'wikipathways_on_PathMe')	2.09389e-15	True
Dataset 2	('reactome_on_PathMe', 'kegg_on_subgraph')	4.67068e-18	True
Dataset 2	('reactome_on_PathMeUniverse', 'reactome_on_subgraph')	7.92979e-14	True
Dataset 2	('reactome_on_PathMeUniverse', 'wikipathways_on_subgraph')	4.39711e-18	True
Dataset 2	('wikipathways_on_PathMeUniverse', 'kegg_on_subgraph')	1.45917e-15	True
Dataset 2	('wikipathways_on_PathMeUniverse', 'reactome_on_subgraph')	4.8137e-18	True
Dataset 2	('wikipathways_on_PathMeUniverse', 'wikipathways_on_subgraph')	5.78237e-16	True
Dataset 2	('kegg_on_subgraph', 'reactome_on_subgraph')	3.89656e-18	True
Dataset 2	('kegg_on_subgraph', 'wikipathways_on_subgraph')	0.0064662	True
Dataset 2	('reactome_on_subgraph', 'wikipathways_on_subgraph')	3.89656e-18	True
Dataset 3	('kegg_on_PathMeUniverse', 'reactome_on_PathMeUniverse')	2.68595e-16	True
Dataset 3	('kegg_on_PathMeUniverse', 'wikipathways_on_PathMeUniverse')	0.318709	False
Dataset 3	('kegg_on_PathMeUniverse', 'kegg_on_subgraph')	3.64168e-17	True
Dataset 3	('kegg_on_PathMeUniverse', 'reactome_on_subgraph')	1.80771e-12	True
Dataset 3	('kegg_on_PathMeUniverse', 'wikipathways_on_subgraph')	8.02384e-18	True
Dataset 3	('reactome_on_PathMeUniverse', 'wikipathways_on_PathMeUniverse')	1.20187e-16	True
Dataset 3	('reactome_on_PathMeUniverse', 'kegg_on_subgraph')	0.000563418	True
Dataset 3	('reactome_on_PathMeUniverse', 'reactome_on_subgraph')	0.584589	False
Dataset 3	('reactome_on_PathMeUniverse', 'wikipathways_on_subgraph')	3.02924e-12	True
Dataset 3	('wikipathways_on_PathMeUniverse', 'kegg_on_subgraph')	1.69063e-17	True
Dataset 3	('wikipathways_on_PathMeUniverse', 'reactome_on_subgraph')	3.50738e-12	True
Dataset 3	('wikipathways_on_PathMeUniverse', 'wikipathways_on_subgraph')	4.26632e-18	True
Dataset 3	('kegg_on_subgraph', 'reactome_on_subgraph')	0.000155475	True
Dataset 3	('kegg_on_subgraph', 'wikipathways_on_subgraph')	7.268e-08	True
Dataset 3	('reactome_on_subgraph', 'wikipathways_on_subgraph')	6.12765e-12	True

Supplementary Figure 14. Wilcoxon test to formalize the differential comparisons of prediction performance of diffusion algorithms in the database validation using AUPRC as a metric. These results can be reproduced with the Jupyter notebook located in the git repository at /evaluations/repeated_holdout/by_database.

4.4.3. Validation by method stratified by -omics modality

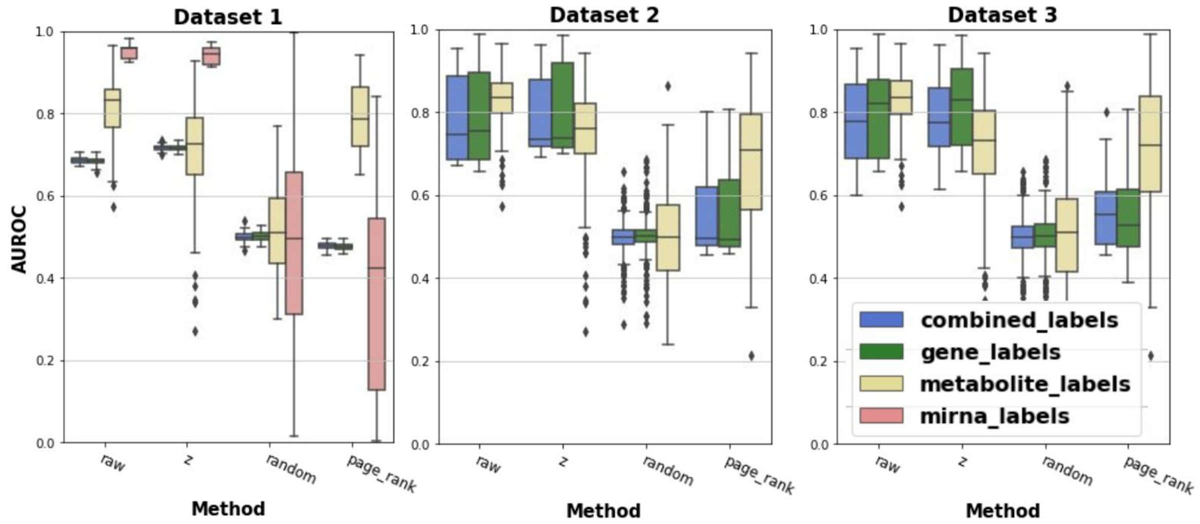

Supplementary Figure 15. Comparison of prediction performance of diffusion algorithms stratified by entity type over the PathMe network and its corresponding subgraphs. Three different multi-omics datasets and various diffusion methods were applied in order to examine the differential influence of each -omics modality compared to the multi-omics integrated graph. The high variability observed at random and PageRank for miRNAs in dataset 1 can be attributed to the low number of entities in that modality. Each box plot shows the distribution of the AUROC over 100 repeated holdout validations. This figure can be reproduced with the Jupyter notebook located in the git repository at /evaluations/repeated_holdout/by_method.

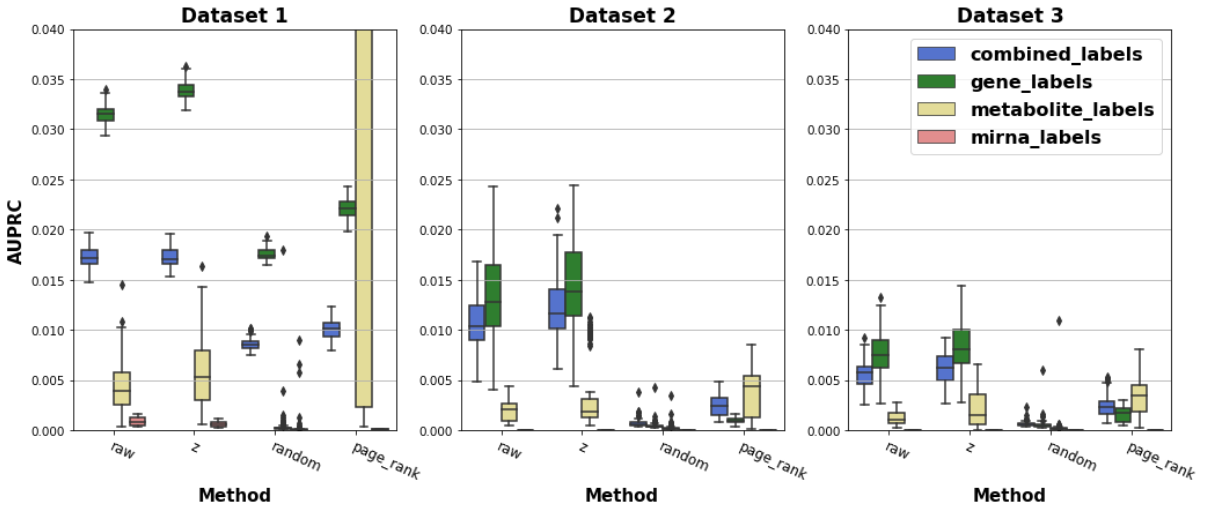

Supplementary Figure 16. Comparison of prediction performance of diffusion algorithms stratified by entity type over PathMe network using three different multi-omics datasets, and applying different diffusion methods, in order to examine the differential partial influence of each -omics modality compared to the multi-omics integrated graph. Each box plot shows the distribution of the AUPRC over 100 repeated holdout validations. This figure can be reproduced with the Jupyter notebook located in the git repository at /evaluations/repeated_holdout/by_method.

4.5. Hardware

Kernel computations were performed on a symmetric multiprocessing (SMP) node with four Intel Xeon Platinum 8160 processors per node with 24 cores/48 threads each (96 cores/192 threads per node in total) and 2.1GHz base / 3.7 GHz Turbo Frequency with 1536GB/1.5TB RAM (DDR4 ECC Reg).

References

1. Ashburner, M., *et al.* (2000). Gene ontology: tool for the unification of biology. The Gene Ontology Consortium. *Nature Genetics,* 25(1), 25–9.

2. Bersanelli, M., *et al.* (2016). Network diffusion-based analysis of high-throughput data for the detection of differentially enriched modules. *Scientific Reports*. (6), 34841.

3. Cowen, L., et al. (2017). Network propagation: a universal amplifier of genetic associations. *Nature Reviews Genetics* 18.9: 551.

4. Denny, J.C, *et al.* (2013). Systematic comparison of phenome-wide association study of electronic medical record data and genome-wide association study data. *Nat Biotechnol.* (12):1102-10.

5. Domingo-Fernández, D., *et al.* (2019). PathMe: Merging and exploring mechanistic pathway knowledge. *BMC Bioinformatics*, 20:243.

6. Fabregat, A., *et al.* (2018). The Reactome pathway knowledgebase. *Nucleic acids research*, *46*(D1):D649-D655.

7. Grüning, B. A., Lampa, S., Vaudel, M., and Blankenberg D. (2019). Software engineering for scientific big data analysis. *GigaScience*, 8(5), giz054.

8. Hagberg, A., Swart, P., and S Chult, D. (2008). Exploring network structure, dynamics, and function using NetworkX. Proceedings of the 7th Python in Science conference (SciPy 2008), G Varoquaux, T Vaught, J Millman (Eds.), pp. 11-15.

9. Harchaoui, Z., *et al.* (2013). Kernel-based methods for hypothesis testing: a unified view. *IEEE Signal Processing Magazine*. (30), 87–97.

10. Huang, H. Y., *et al.* (2020). miRTarBase 2020: updates to the experimentally validated microRNA–target interaction database. *Nucleic acids research*, *48*(D1), D148-D154.

11. Kanehisa, M., *et al.* (2016). KEGG: new perspectives on genomes, pathways, diseases and drugs. *Nucleic acids research*, *45*(D1), D353-D361.

12. Kuhn, M., *et al.* (2016). The SIDER database of drugs and side effects. *Nucleic Acids Research*, 44(D1), D1075–D1079.

13. Menche, J., *et al.* (2015). Disease networks. Uncovering disease-disease relationships through the incomplete interactome. *Science,* 347(6224), 1257601.

14. Mostafavi, S., *et al.* (2008). Genemania: a real-time multiple association network integration algorithm for predicting gene function.*Genome Biology.* (9), S4.

15. Murakami, Y., *et al.* (2015). Comprehensive analysis of transcriptome and metabolome analysis in Intrahepatic Cholangiocarcinoma and Hepatocellular Carcinoma. *Scientific reports*, *5*(1), 1-12.

16. Picart-Armada, S., *et al.* (2017). diffuStats: an R package to compute diffusion-based scores on biological networks. *Bioinformatics*, 34(3), 533-534.

17. Picart-Armada, S., *et al.* (2017). Null diffusion-based enrichment for metabolomics data. *PloS one*, 12(12).

18. Ren, S., *et al.* (2016). Integration of metabolomics and transcriptomics reveals major metabolic pathways and potential biomarker involved in prostate cancer. *Molecular & Cellular Proteomics*, *15*(1), 154-163.

19. Slenter, N., *et al.* (2017). WikiPathways: a multifaceted pathway database bridging metabolomics to other omics research. *Nucleic Acids Research*., 46(D1), D661-D667.

20. Smola, A. J., Kondor, R. (2003). Kernels and regularization on graphs. *Learning theory and kernel machines (pp. 144-158).*

21. Tsuda, K., *et al.* (2005). Fast protein classification with multiple networks. *Bioinformatics*, (21), 59–65.

22. Valentini, G., *et al.* (2016). RANKS: a flexible tool for node label ranking and classification in biological networks. *Bioinformatics*, 32(18), 2872-2874.

23. Van den Hof, W. F., *et al.* (2015). Integrating multiple omics to unravel mechanisms of Cyclosporin A induced hepatotoxicity in vitro. *Toxicology in Vitro*, *29*(3), 489-501.

24. Vandin, F., *et al.* (2010). Algorithms for detecting significantly mutated pathways in cancer. *Lecture Notes in Computer Science.* 6044, 506–521.

25. Wishart, D. S., *et al.* (2018). DrugBank 5.0: a major update to the DrugBank database for 2018. *Nucleic Acids Research*, 46(D1), D1074–D1082.

26. Yen, L., et al (2007). Graph nodes clustering based on the commute-time kernel. *Pacific-Asia Conference on Knowledge Discovery and Data Mining 2007*. p. 1037-1045.

27. Zhou, X., Menche, J., Barabási, A. L., and Sharma, A. (2014). Human symptoms–disease network. *Nature communications*, *5*(1), 1-10.
